# Vulnerable consumers: marketing research needs to pay more attention to the brain health of consumers

**DOI:** 10.1007/s11002-022-09654-3

**Published:** 2022-11-03

**Authors:** Andrija Javor, Monika Koller, Nick Lee, Hans Breiter

**Affiliations:** 1 Ronin Institute, Montclair, NJ 07043 USA; 2 Department of Marketing, Institute for Marketing and Consumer Research, WU Vienna University of Economics and Business, Welthandelsplatz 1, 1020 Vienna, Austria; 3 Warwick Business School, University of Warwick, Coventry, CV4 7AL UK; 4 Department of Psychiatry and Behavioral Sciences, Northwestern University, Chicago, IL USA; 5 Laboratory of Neuroimaging and Genetics, Department of Psychiatry, Massachusetts General Hospital and Harvard School of Medicine, Boston, MA USA

**Keywords:** Vulnerable consumers, Research methods, Neuroscience, Brain health, Causal inference, Marketing ethics

## Abstract

We propose here that marketing research should increase consideration of the brain health of consumers, and argue that it would help both extend our current knowledge of vulnerable and other marginalised groups, as well as extend generalizability and external validity of marketing research in general. We show that such a focus would help enrich methodology, especially around causal inference, as well as impact on our understanding of a number of key emerging themes in marketing research. We particularly focus on the consumer behaviour around digitalisation, as well as compulsive buying behaviour. Further, we show that increasing consideration of consumer brain health will further efforts towards inclusivity of marketing, and help continue progress towards marketing research as a force for good.

A major boundary condition of existing marketing knowledge is that it is primarily based on homogenous study populations, which typically exclude marginalised consumers such as special need populations and vulnerable consumer groups. A vulnerable consumer can be defined as a consumer who, as a result of socio-demographic/behavioural characteristics, personal situation, or market environment, is at higher risk of experiencing negative outcomes in the market, has limited ability to maximise his/her well-being, has difficulty in obtaining or assimilating information, is less able to buy, choose or access suitable products, or is more susceptible to certain marketing practices (Jourova, 2016). Although the topic of consumer vulnerability has gained some attention in marketing research over the last decade (e.g. Pechmann et al., 2011), we focus specifically here on vulnerability due to the *brain health* status of consumers. This topic has so far not been a focus of marketing research. In the following paragraphs, we will argue that demographic changes and an increase in prevalence of disorders and other impairments of brain health require a more inclusive approach in future research.

The term “brain health” (as opposed to “mental health”) refers to the preservation of optimal brain integrity and mental and cognitive functions, combined with an absence of neurological and psychiatric disorders (Wang et al., 2020), such as depression, anxiety, dementia, epilepsies, and Parkinson’s disease. Brain health has been a focus of several research fields for many years but has recently received increasing societal attention due to current developments, such as the aging population, associated with an increase in neurological and psychiatric diseases such as stroke and dementias (Feigin et al., 2019), as well as the SARS-COV2 pandemic and related neuropsychiatric sequelae (Taquet et al. 2021). However, apart from research on maladaptive consumption behaviour (e.g. Reimann & Jain, 2021), the brain health status of consumers has been neglected in marketing research. In this idea corner paper we call for marketing research, if it wishes to make a genuine positive impact on a better world, to increase focus on the brain health of consumers. Specifically, we believe that a focus on consumer brain health and associated phenomena is vital for future marketing science, since (1) brain health is affected in a significant and increasing fraction of consumers (Feigin et al., 2019), (2) impairments of brain health can specifically affect consumer behaviour and/or marketplace phenomena (e.g. Bani-Rshaid & Alghraibeh, 2017), and (3) inclusion of consumers that are vulnerable due to their brain health status may open up another way to establish causality in marketing.

## Marketing research and consumer brain health

The research agenda we propose consists of four broad themes where a focus on brain health can have a major impact. Taking in aspects of methodology, emerging as well as established topics, we hope to inspire researchers to consider brain health-related issues in their own work.

1. *Impact on our understanding of methodological approaches in marketing research*
  Making causal inferences is a primary goal in marketing (Varian, 2016). Although experiments are valuable tools in this regard, they remain subject to noise of several kinds. Adding insights regarding brain health would significantly enhance knowledge regarding a multitude of psychological phenomena. For example, considering brain health enhances causality by demonstrating that suppressed activation in relevant corresponding brain areas (in diseased patients) directly affects the ability to exhibit related behaviour. This approach goes beyond our currently used tool-box in marketing. Clinical science in medicine traditionally generates knowledge about a disease by a comparison of a certain variable (such as behaviour) between healthy and diseased subjects that are otherwise comparable (or matched, such as with demographic variables), so that any difference is more likely to be causally linked to the disease or affected anatomical areas.
  Focusing on brain health issues then does not only enrich our understanding of marketing topic-wise but also opens another way to establish causality. Do our theories and models that we have generated over the past decades hold for these vulnerable consumer groups? How can a brain health perspective further enrich our methodology used? How does it integrate into currently heavily studied topics such as machine learning, AI, and neuroscience?
2. *Impact on our understanding of digitalisation in marketing*
  Digital solutions are one key element of contemporary marketing. It is important to sensitise readers to existing evidence suggesting differences in behaviour between healthy consumers and people with brain diseases in human–computer interaction that might impact digital marketing. As an example, Javor et al. (2016) used a trust game paradigm to simulate social behaviour between Parkinson’s disease patients (experimental group) or healthy participants (controls) with human or avatar characters on a website. The concept behind this experiment follows the above-mentioned clinical approach, which stipulates that, given two groups that are comparable in all other variables such as age, education, or income, any behavioural difference must be explained by the disease process itself. Parkinson’s patients showed reduced trust in simulated interactions with humans, whilst trust behaviour in interactions with avatars did not differ significantly from controls. Hence, whilst low trust might lead to a vulnerability of Parkinson patients in interpersonal interactions, this might not be the case in interactions with avatars. This has potential implications for the design of marketing campaigns or websites that warrant future research. Other questions for future research could be: What does it mean to optimise user experience and thereby augment accessibility of computers in a vulnerable consumer population? What does engaging with new web technology (e.g. the Metaverse) mean for consumers suffering from certain diseases that lead to differences in trust and other behaviours between human–human and human-avatar interactions?
3. *Impact on our understanding of consumer behaviour*
  A number of areas of consumer behaviour could benefit from focusing on vulnerability due to brain health status. One example is pro-social behaviour. Javor et al. (2019) found that patients with a form of epilepsy show increased pro-social behaviour compared to healthy controls. This finding links to differences in activation of parts of the reward system, which is known to play a crucial role in consumer behaviour. Are these patients at greater risk of being influenced by others due to their overall increased pro-social behaviour? Do they exhibit more pro-social purchase behaviour involving self-sacrifice for the good of others or society (Small & Cryder, 2016)? What implications does this have for marketing communications addressed at vulnerable consumers (e.g. in a way to reduce activation of the reward system) in order to be protective? Should product design and pricing be considered for customers vulnerable due to their brain health status?
4. *Impact on our understanding of marketing as a force for good — inclusion marketing and ethics*
  Focusing on the brain health of consumers has the potential to reshape marketing thought and practice by altering how we conduct research, derive implications, and how we communicate with consumers. It is a chance to reshape marketing and to use marketing as a force for good.
  Furthermore, marketing research has an obligation to contribute to academia, practice, and policy makers and society. If the brain health of consumers is to be incorporated correctly into marketing research, the relevant guidelines and rules of clinical research (e.g. the World Medical Association Declaration of Helsinki in 2015) should thus be tested for relevance in marketing research practices. Furthermore, marketing-related ethical considerations should be added to this perspective, as most would agree that certain customer populations, such as ill, disabled, or disadvantaged/powerless individuals, need special protection and that biological disorders must not be misused by being specifically targeted by marketing activities.
  We already observe such a shift in priorities in the editorial scopes of academic marketing journals as well as in more interdisciplinary collaborations (e.g. the “Better Marketing for a Better World”-initiative), and this presents opportunities for marketing scholars to ask intriguing research questions: What does marketing as a force for good mean from the perspective of the brain health of consumers? How do we manage to foster an including rather than excluding approach in marketing? What does this mean for companies, for our understanding of brand management, customer experience, or digital targeting in marketing communications?

## Conclusion

We live today in a world replete with grand health challenges of which many have become directly or peripherally associated with marketing practice. Although we have acknowledged vulnerability of consumers in terms of brain health, our research has only marginally adopted a perspective that allows for testing whether the findings published in the top journals of our field hold for these consumers as well. Performing experimental research to understand the consequences of brain pathology of neurological and psychiatric patients on classical marketing constructs, such as purchase intention and behaviour, will clarify in what aspect this group is vulnerable and what targeted protection measures are needed. Such an approach would help to balance the need for specific protections on the one hand, and stigmatisation of patients as being “generally” vulnerable and “unable” to perform purchasing acts without assistance on the other.
